# Longitudinal Analysis of Circulating Tumor Cells in Colorectal Cancer Patients by a Cytological and Molecular Approach: Feasibility and Clinical Application

**DOI:** 10.3389/fonc.2021.646885

**Published:** 2021-06-28

**Authors:** Alexander Hendricks, Katharina Dall, Burkhard Brandt, Reinhild Geisen, Christian Röder, Clemens Schafmayer, Thomas Becker, Sebastian Hinz, Susanne Sebens

**Affiliations:** ^1^ Department of General, Visceral, Thoracic, Transplantation and Pediatric Surgery, University Hospital Schleswig-Holstein Campus Kiel, Kiel, Germany; ^2^ Institute of Clinical Chemistry, University Hospital Schleswig-Holstein Campus Kiel, Kiel, Germany; ^3^ ORGA Labormanagement GmbH, Ochtrup, Germany; ^4^ Institute for Experimental Cancer Research, Kiel University and University Hospital Schleswig-Holstein Campus, Kiel, Kiel, Germany

**Keywords:** circulating tumor cells, colorectal cancer, NYONE^®^ cell imager, CK20 RT-qPCR, longitudinal follow-up, liquid biopsies

## Abstract

**Introduction:**

Liquid biopsies allowing for individualized risk stratification of cancer patients have become of high significance in individualized cancer diagnostics and treatment. The detection of circulating tumor cells (CTC) has proven to be highly relevant in risk prediction, e.g., in colorectal cancer (CRC) patients. In this study, we investigate the clinical relevance of longitudinal CTC detection over a course of follow-up after surgical resection of the tumor and correlate these findings with clinico-pathological characteristics.

**Methods:**

In total, 49 patients with histologically proven colorectal carcinoma were recruited for this prospective study. Blood samples were analyzed for CTC presence by two methods: first by marker-dependent immunofluorescence staining combined with automated microscopy with the NYONE^®^ cell imager and additionally, indirectly, by semi-quantitative Cytokeratin-20 (CK20) RT-qPCR. CTC quantification data were compared and correlated with the clinico-pathological parameters.

**Results:**

Detection of CTC over a post-operative time course was feasible with both applied methods. In patients who were pre-operatively negative for CTCs with the NYONE^®^ method or below the cut-off for relative CK20 mRNA expression after analysis by PCR, a statistically significant rise in the immediate post-operative CTC detection could be demonstrated. Further, in the cohort analyzed by PCR, we detected a lower CTC load in patients who were adjuvantly treated with chemotherapy compared to patients in the follow-up subgroup. This finding was contrary to the same patient subset analyzed with the NYONE^®^ for CTC detection.

**Conclusion:**

Our study investigates the occurrence of CTC in CRC patients after surgical resection of the primary tumor and during postoperative follow-up. The resection of the tumor has an impact on the CTC quantity and the longitudinal CTC analysis supports the significance of CTC as a prognostic biomarker. Future investigations with an even more extended follow-up period and larger patient cohorts will have to validate our results and may help to define an optimal longitudinal sampling scheme for liquid biopsies in the post-operative monitoring of cancer patients to enable tailored therapy concepts for precision medicine.

## Introduction

Despite tremendous efforts in the diagnosis and treatment of colorectal cancer (CRC), it still represents one of the most common causes of cancer-related deaths in Western countries (1). The fact that a proportion of patients is diagnosed with a localized tumor that can be resected in sano (R0) but later develop a tumor recurrence or distant metastases underlines the need for valid prognostic and predictive biomarkers that help to identify high-risk patients. Profound criteria for the stratification of patients at risk who might benefit from an adjuvant treatment have been developed (2, 3), though these almost all rely on histopathological parameters amongst very few other mutational characteristics of the primary tumor.

Consequentially, the concept of individualized diagnostics and therapeutic options has yielded major attention in recent years (4, 5), and biomarkers for either early detection of cancer or proof of minimal residual disease have been identified (6). As a potential tool, circulating tumor cells (CTC) have been identified and their suitability to serve as an additional instrument in risk stratification has been demonstrated manifold (7). These CTC are shed into the peripheral bloodstream not only from the primary but also from metastatic tumor sites and are linked to progressive disease and metastatic formation. In most cases of CRC patients with local disease, tumor resection is considered as a curative approach. The impact of surgery on the CTC count in the bloodstream has been already described, with generally a steep increase in CTC numbers shortly after surgical resection, but also a rapid normalization and often decrease in cell numbers within a short period of time (8). Though, studies on the enumeration of CTC in the long-term longitudinal follow-up of patients with solid tumors after surgery are rare.

CTC are extremely rare in the bloodstream and their valid detection and enumeration amongst multifarious numbers of leukocytes pose a major challenge. Up to date, various enrichment and detection techniques are available (9, 10). Categorically, CTC can be directly detected and enumerated by the means of cytological immunological staining, or indirectly detected by molecular approaches using PCR. The cytological approach for CTC enumeration is mostly marker-dependent, though many techniques employ only single antibodies for visualization of CTC. In this context, the most commonly used target antigens are EpCAM or several cytokeratins, which are highly specific to CTC of epithelial tumors. For the molecular detection of CTC, we previously reported on an RT-qPCR against cytokeratin 20 (CK20), which was already established to determine the prognostic value of the CTC load at the time point of surgery in respectable CRC patients (11, 12). By the introduction of a cut-off value allowing a relative CTC quantification, the negative prognostic significance of the amount of CK20-positive CTC in CRC patients could clearly be demonstrated (13).

In order to further extend these findings and to validate our CTC detection approach, this study aimed at a proof-of-principle study for a longitudinal follow-up of CRC patients after surgical resection with a series of set timepoints for blood draw. Furthermore, both a novel immunofluorescence-based and a molecular detection approach for enumeration and detection of CTC was employed and the results of both methods were compared. For both detection methods, peripheral blood mononuclear cells (PBMC) were enriched by density gradient centrifugation (Ficoll^®^ or CPT Vacutainer) and then applied in either analysis For enumeration by the semi-automated cell-imager (NYONE^®^, SYNENTEC, Elmshorn, Germany) CTC were immunofluorescently (IF) stained utilizing a set of antibodies against highly specific antigens of CTC in CRC patients, namely, anti-EpCAM, anti-EGFR, anti-pan-Cytokeratin(CK), and anti-Her2, as established in an earlier study (14). Additionally, an established CK20 RT-qPCR assay was applied for relative CTC quantification, as described elsewhere (13). The obtained data were then correlated to clinical characteristics and follow-up records, e.g., local recurrence, adjuvant treatment. Special emphasis was laid on the longitudinal postoperative CTC detection since individual therapeutic decisions are frequently made based on the histopathological characterization of the tumor at the time of primary surgery.

## Materials and Methods

### Patient Recruitment and Serial Sampling

In total, 49 patients with a histologically verified CRC were enrolled in this prospective study in the years 2017 and 2018. All patients were operated on at the Department of General, Visceral, Thoracic, Transplantation and Paediatric Surgery of the University Hospital Schleswig-Holstein (UKSH), Campus Kiel. In case staging diagnostics of a rectal carcinoma revealed a locally progressed tumor burden with either T3/T4 and/or N+ according to the TNM classification (TNM Classification of Malignant Tumors eighth edition), patients were admitted to a neoadjuvant radio-chemotherapy (RCTX). Patients, who were staged UICC (Union internationale contre le cancer) III or IV after histopathological examination were recommended to be admitted to either adjuvant or palliative chemotherapy (CTX) post-operatively. All decisions were made according to the present guidelines (German S3-Guideline Colorectal Carcinoma, Version 2.1 – January 2019 AWMF-Registration Number: 021/007OL) and the general patients´ constitution in terms of morbidity and endorsement. All patients gave written informed consent to participate in this study. The study was approved by the local ethics committee of the UKSH Campus Kiel and the Medical Faculty, University of Kiel (#A110/99). Classification of the pathological tumor stage was handled by the Department of Pathology, UKSH Campus Kiel, according to the TNM-classification. Clinical data were obtained from the clinical research database of the oncological biobank of the Comprehensive Cancer Center Kiel (BMB-CCC) and data was verified by re-examination of original patient records.

**Figure 1:**
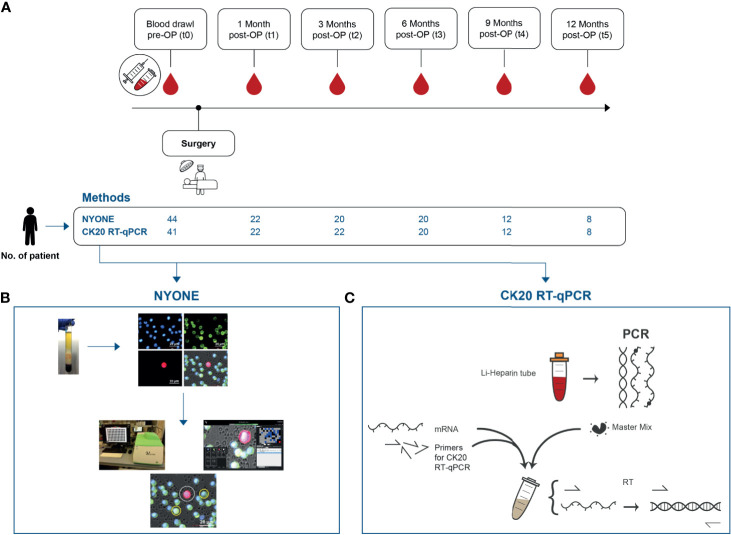
Study outline and synopsis of the employed techniques for CTC detection. **(A)** Blood samples were collected repeatedly over a course of 12 months post-operatively. The first blood sample of each recruited patient was drawn immediately pre-operatively t0. Over the study period, five more samples were collected at set follow-up visits of each patient at 4 weeks (t1), 3 months (t2), 6 months (t3), 9 months (t4), and 12 months (t5). **(B)** NYONE^®^ – After CTC enrichment by Ficoll centrifugation *via* CPT tubes, PBMC were fixed, permeabilized, and stained with anti-CD45-Alexa488 (green to detect leukocytes), anti-EpCAM, anti-EGFR, anti-Her2, and anti-pan-CK antibodies (all Alexa647-coupled, red to detect epithelial cells) and DAPI (blue) for nuclei staining. The enumeration process of CTC was carried out by the cell imager NYONE^®^. After image analysis, CTC (DAPI positive, negative for Alexa488, and positive for Alexa647) were encircled allowing cytological assessment. **(C)** Semi-quantitative RT-qPCR against CK20 –After Ficoll centrifugation of the blood plasma samples, the enriched fraction of PBMC was isolated and cDNA was obtained after RNA preparation. Then, a TaqMan gene expression assay [KRT20 (CK20)] and TBP as a house-keeping gene were used.

Blood samples from the following time points were analyzed for the presence of CTC: Pre-operatively (t0) and the primary endpoint of the study was reached if a patient reached the last blood draw at 12 months post-operatively (t5). For this, each patient received an individualized follow-up regimen for a visit and blood sample drawing at set time points after the surgical procedure: one month (t1), three months (t2), 6 months (t3), 9 months (t4), and 12 months (t5) (**Figure 1A**). The peripheral blood samples were either taken shortly prior to surgery (t0) from a central venous line or obtained by puncture of the median cubital vein for the blood samples collected at the follow-up time points (t1-t5).

For this study, two different approaches for CTC detection were applied. For the immunofluorescence detection by NYONE^®^, approximately 8 ml of blood were collected into a Sodium Citrate-Monovette^®^ (Sarstedt, Nümbrecht, Germany). For CTC detection by PCR, approximately 20 ml of blood were drawn into lithium heparin-Monovettes^®^ (Sarstedt). All samples were further processed within 2 hours after blood draw.

### Sample Analysis With the Semi-Automated Microscope—NYONE^®^

The establishment and procedure of CTC enumeration by semi-automated microscopic detection with the cell imager NYONE^®^ (SYNENTEC, Elmshorn, Germany) has been described previously (14). Briefly, the mononuclear cell (MNC) fraction was isolated by Ficoll-cushion centrifugation and resuspended in a fixation buffer (#14190-094, Biolegend, San Diego, CA, USA), incubated for 15 minutes at room temperature (RT), after which the samples were stored at 4°C for up to four days until further analysis.

Then, cells were permeabilized in a Perm-/Wash-Buffer (#421002, Biolegend) for 5 minutes at RT and centrifuged at 330xg for 10 minutes. Afterward, cells were incubated with an Fc-blocking buffer (#422301/2, Biolegend) for 15 minutes. Then, cells were incubated for 30 minutes with the following antibodies: anti-CD45-AF488 (#304017; Biolegend), anti-EpCAM-AF647 (#324212; Biolegend), anti-pan-CK-AF647 (#628604; Biolegend), anti-EGFR-AF647 (#sc-120 AF647; SantaCruz, Dallas, TX, USA), and anti-Her2-AF647 (#3244412; Biolegend). Finally, a buffer containing DAPI (1:10,000) (#422801; Biolegend) was added to the cells.

Subsequently, 200 µl of the stained cell suspension was transferred into each a well of two 96-well plates (Sarstedt), which was centrifuged at 330xg for 10 minutes, and afterward placed in the NYONE^®^ cell imager.

For analysis, the plates were scanned only for the detection of Alexa647 fluorescence (Ex 632/22, Em 685/40) as only CTC should be positive in this setting, which was detected by the respective antibody cocktail against epithelial markers EpCAM, pan-cytokeratin, EGFR, HER2. SYNENTEC’s proprietary YT^®^-Software automatically analyzed the images already during scanning and detected positive events (**Figure 1B**). The image processing settings for this analysis were determined previously using blood samples from healthy donors spiked with HT29 tumor cells (14). Each event was then automatically further analyzed in depth by creating a region of interest (ROI) around it. This ROI was scanned in four channels (DAPI: Ex 377/50 Em 452/45, Alexa488: Ex 475/28 Em 530/43, Alexa-647: Ex 632/22 Em 685/40, brightfield: Ex brightfield Em blue). YT^®^-Software then automatically detected the cells´ nuclei (DAPI, blue fluorescence) and analyzed whether a virtual cytoplasm surrounding the nuclei was fluorescing green (CD45) or red (EpCAM, pan-CK, EGFR, Her2). These events were finally presented separately by the software and the investigator was able to examine the morphology of the potential CTC (**Figure 1B**).

### Sample Analysis by Molecular mRNA Detection: Semi-Quantitative CK20 RT-qPCR

The application of a semi-quantitative CK20 RT-qPCR for CTC detection (**Figure 1C**) in CRC patients has been previously established in our work group (13). Briefly, blood samples were processed by ficoll-centrifugation to isolate the MNC fraction. Then, RNA was isolated with RNAPure^®^ reagent (VWR Peqlab, Darmstadt, Germany) and cDNA was obtained by reverse transcription of 3 μg total RNA (Maxima First Strand cDNA Synthesis Kit, Thermo Fisher Scientific, Darmstadt, Germany). The qPCRs were run in a total volume of 20 μl per well of a 96-well plate (Sarstedt) using the TaqMan gene expression assays for CK20 (KRT20, Hs00966063_m1) and for the housekeeping gene TBP (TATA-box binding protein), Hs00427620_m1, as a reference in combination with the TaqMan Fast Advanced Master Mix on a StepOne Plus realtime PCR System (all ThermoFisher Scientific). All samples were run in triplicate. Relative gene expression was calculated as arbitrary expression units [EU] by a simplified ΔC_t_ method normalizing the CK20 expression against the reference gene TBP expression.

### Statistical Analysis

All reported P-values are two-sided and were regarded statistically significant at P < 0.05. When a Gaussian distribution of the data was assumed, the parametric data were analyzed by either a repeated measure or ordinary one-way-ANOVA test. Non-parametric data were analyzed by a Mann-Whitney U-test. Statistical calculation and testing were performed with GraphPad Prism 8 (GraphPad Software, San Diego, CA, USA).

## Results

### Patients Demographics

A synopsis of all patient data relating to the entire cohort but also the subgroups in terms of detection method is given in **Table 1**. In total, blood samples from 49 patients were included in this prospective study (**Figure 1A**). A total of 30 male and 19 female patients were enrolled and the median age at the time of operation and first blood sample collection was 67 years (range: 48–89 years). In total, 32 patients were diagnosed with colon carcinoma and 17 patients with rectal carcinoma. Amongst the colon carcinoma subset, an equal composition between left- and right-sided carcinoma (both 16 cases) was noted. The study cohort was further stratified by a clinico-pathological staging according to the UICC stages I-IV, with the most patients diagnosed with stage III (38.8%). In total, 10 patients (all rectal carcinoma) were treated by neoadjuvant radio-chemotherapy, and 18 patients received adjuvant chemotherapy.

**Table 1:** Patient demographics and clinical characteristics of the entire study population and further breakdown according to the utilized detection modes.

	Total *N* (%)	NYONE *N* (%)	CK20 *N* (%)
	49 (100)	44 (100)	47 (100)
**Gender**			
Male	30 (61.2)	26 (59.1)	29 (61.7)
Female	19 (38.8)	18 (40.9)	18 (38.8)
**Age**			
Median (range)	67 (45-89)	66 (45-89)	67 (45-89)
<65	21 (42.9)	18 (40.9)	21 (44.7)
≥65	28 (57.1)	26 (59.1)	26 (55.3)
**Tumor site**			
Colon	32 (65.3)	32 (72.7)	30 (63.8)
Right	16 (50.0)	16 (50.0)	15 (50.0)
Left	16 (50.0)	16 (50.0)	15 (50.0)
Rectum	17 (34.7)	12 (27.3)	17 (36.2)
**UICC stage**			
I	14 (28.6)	12 (27.3)	14 (29.8)
II	9 (18.4)	9 (20.5)	8 (17.0)
III	19 (38.8)	18 (40.9)	18 (38.3)
IV	7 (14.3)	5 (11.4)	7 (14.9)
**Neoadj. treatment**			
Yes	10 (58.8)	6 (50.0)	10 (58.8)
No	7 (41.2)	6 (50.0)	7 (41.2)
**Adjuvant treatment**			
Yes	18 (36.7)	14 (31.8)	17 (36.2)
No	31 (63.3)	30 (68.2)	30 (63.8)

The subset of patients who received neoadjuvant treatment solely comprised of patients with rectal carcinoma. UICC, Union internationale contre le cancer.

Altogether, 44 and 47 patients were enrolled for CTC analysis by the cytological semi-automated microscopy (NYONE^®^, **Figure 1B**) and the indirect molecular approach by CK20 RT-qPCR (**Figure 1C**), respectively. Generally, the distribution of the two subsets of patients according to the demographical and clinical parameters was assimilable. The median age of patients within the NYONE^®^ subgroup was 66 years (range: 45–89 years) and within the PCR group 67 years (range: 45–89 years) at the time of blood draw. In both groups, the majority of patients were male (59.1% – NYONE^®^ and 61.7% – PCR) and were diagnosed with a colon carcinoma (72.7% – NYONE^®^ and 63.8% – PCR). Again, most of the patients were diagnosed with locally advanced tumor burden and staged UICC III (40.9% – NYONE^®^ and 38.3% PCR).

### Longitudinal Analysis of CTC Count by IF and the NYONE^®^ Cell-Imager

Altogether, we were able to enroll 44 patients for the longitudinal follow-up. During the time of the study period, the number of patient re-visits declined (**Figure 1A**).

In general, positivity rates during the collection time-course of CTC by the NYONE^®^ technique were low and comparable at the first (pre-operative, t0) time points (**Table 2**), ranging between a mean CTC count of 0.89 and 1.5. At t5, a considerable increase of the mean CTC count could be observed (mean 4.25 CTC; SD: 10.01). However, this might be explained by one patient´s exceedingly high CTC count of 29 IF-positive cells.

**Table 2:** CTC quantity partitioned for each technique of CTC detection and outlined for each follow-up timepoint with association of the impact of adjuvant chemotherapy on the CTC quantity over the study period.

	t0	*p*	t1	*p*	t2	*p*	t3	*p*	t4	*p*	t5	p
**NyOne**												
CTC positive patients: fraction (%)	16/44 (36.4)		12/22 (54.5)		12/22 (54.5)		9/20 (45.0)		7/12 (58.3)		6/8 (75.0)	
CTC count per patient: mean (SD)	0.89 (1.57)		1.18 (1.33)		1.50 (2.61)		1.00 (1.69)		1.33 (1.67)		4.25 (10.01)	
CTC count according to adjuvant chemotherapy												
CTX+: mean (SD)	1.50 (2.28)	ns	2.60 (1.34)	ns	2.25 (3.28)	ns	0.86 (0.90)	ns	0.83 (0.98)	ns	7.75 (14.17)	**0.015**
CTX-: mean (SD)	0.60 (1.04)		0.76 (1.03)		1.07 (2.16)		1.08 (2.02)		1.83 (2.14)		0.75 (0.50)	
**CK20 RT-qPCR**												
CTC positive patients: fraction (%)	33/41 (80.5)		19/22 (86.4)		17/22 (77.3)		16/20 (80.0)		7/12 (58.3)		8/8 (100.0)	
CTC count per patient: mean (SD)	3.11 (3.81)		3.55 (6.17)		3.08 (3.19)		2.41 (1.62)		1.61 (1.95)		4.16 (5.66)	
Patients ≥ cut-off: fraction (%)	15/41 (36.6)		8/22 (36.4)		11/22 (50.0)		10/20 (50.0)		2/12 (16.7)		3/8 (37.5)	
CTC count according to adjuvant chemotherapy												
CTX+: mean (SD)	2.43 (2.42)	ns	2.00 (1.85)	ns	3.21 (3.98)	ns	1.74 (1.45)	ns	1.21 (1.49)	ns	1.86 (0.55)	ns
CTX-: mean (SD)	3.40 (4.26)		4.14 (7.13)		3.00 (2.81)		2.76 (1.64)		2.01 (2.41)		6.45 (7.77)	

All p values in bold are regarded as statistically significant. UICC, Union internationale contre le cancer; SD, standard deviation; CTC, Circulating tumor cells; CTX, chemotherapy; ns, not significant.

**Figure 2:**
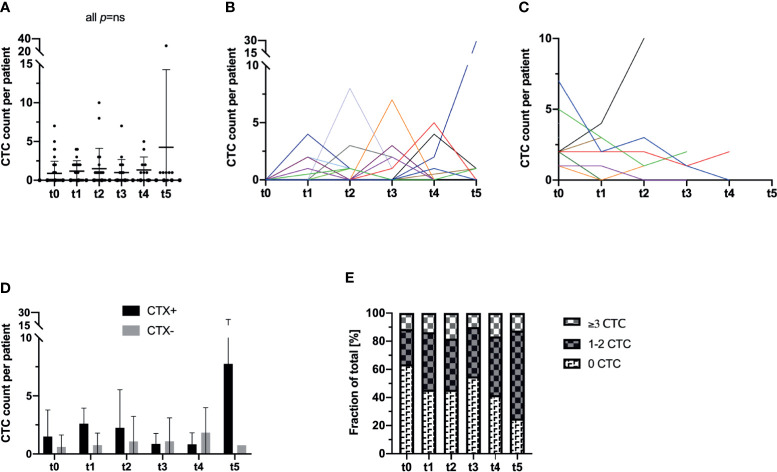
Longitudinal analysis of blood samples of 44 CRC patients for the incidence and enumeration of CTC by a semi-automated microscopical approach with NYONE^®^. **(A)** In the study cohort as a whole, no statistically significant deviations in terms of an in- or decrease of the CTC count compared to t0 could be observed. The bar represents the mean count of CTC. **(B, C)** The individual patient with its longitudinal CTC quantification data is displayed by each line (each color represents one patient). **(B)** All patients that pre-operatively (t0) had no detectable CTC were analyzed in this subset. At t1, there was a statistically significant (p=0.023) increase in the CTC quantity. Throughout the further visits, no significant deviation from the initial CTC quantity (t0) was observed. **(C)** All patients with detectable CTC at t0 were sub-grouped for this analysis. There was no statistical significance for deviations over the study period from the initial CTC count. **(D)** Patients were stratified and subdivided according to their necessity of adjuvant chemotherapeutic treatment (CTX+). Patients that did not require adjuvant treatment were grouped in the follow-up subset (CTX-). **(E)** Patients were stratified and grouped according to the patients´ individual quantity of CTC: No CTC, 1-2 CTC (intermediate), ≥3 CTC (high). Analyzing the data as fractions of a whole, throughout the study a significant increase of patients with intermediate or high CTC counts was monitored.

Surgical resection of the tumor did not seem to have an effect on the frequency of CTC in the peripheral blood of the patients as the mean count of CTC was 0.89 CTC (range: 0–7 CTC; SD: 1.57) at t0 (prior to surgery) and 1.18 CTC (range: 0–4 CTC; SD: 1.33) at t1 (p not significant). Furthermore, analyzing the following blood samples over the time course, the CTC count of the overall study population did not show any significant alterations from the initial CTC prevalence (all p not significant) (**Table 2** and **Figure 2A**).

Stratifying the study population by means of CTC positivity (n=16 patients) *vs*. negativity (n=28 patients) at t0, a statistically significant increase in the CTC count at t1 was monitored in the subgroup without any pre-operative signs of CTC (mean: 0.00; SD: 0.00 at t0 and mean: 0.93; SD: 1.22 at t1; p=0.023). In all other measurements at later time points of the study, no significant differences compared to the baseline at t0 were observed in this subgroup (all p not significant) (**Figure 2B**). In contrast, in patients who initially had shown evidence of CTC in the peripheral blood, a general decrease in CTC by trend could be monitored (**Figure 2C**).

Next, we further stratified the cohort by adjuvant chemotherapeutic treatment and analyzed patients who received treatment (CTX+) in comparison to patients who were solely admitted to follow-up care (CTX-). We analyzed the patients´ CTC counts accordingly and compared the mean cell counts of CTC at each time point individually. Surprisingly, CTX+ patients showed higher CTC counts almost throughout the entire study period with a statistically significant higher CTC amount at t5 (CTX+: mean 7.75 cells, SD 14.17 *vs*. CTX-: mean 0.75 cells, SD 0.5; p=0.015) (**Table 2** and **Figure 2D**).

We additionally analyzed the development of the cohort during the observation period based on the amount of CTC. Subgroups were defined by the absence of CTC (0 cells per patient sample), intermediate frequency (1–2 cells per patient sample), and high CTC rates (≥3 cells per patient sample). The definition of high CTC frequency as ≥3 cells was based on different studies which proposed this as a clinically significant cut-off determined by the CellSearch^®^ system (15). Interestingly, while the percentage of patients with no cells in the blood declined during the time period of the study, a highly significant increase in patients with both intermediate (1–2 CTC) and high prevalence of CTC (≥3 CTC) was monitored for the duration of the study (p=0.002) (**Figure 2E**). In detail, at t0 approximately 63.3% of the patients were CTC negative while at t5 the percentage declined to 25.0%.

### Longitudinal Analysis of the Relative CTC Load by CK20 RT-qPCR

In total, 47 patients were recruited in this study arm. **Table 1** gives an overview of the clinical and pathological data of the patients. Blood samples that were collected at the time of operation and further samples that allowed for longitudinal CTC analysis were available from 41 patients.

In terms of tumor stages, the present patient cohort is representative, and the sensitivity rate of our applied CK20 RT-qPCR (80.42% positive for CTC, **Table 2**) is comparable to our previously reported data (13).

**Figure 3:**
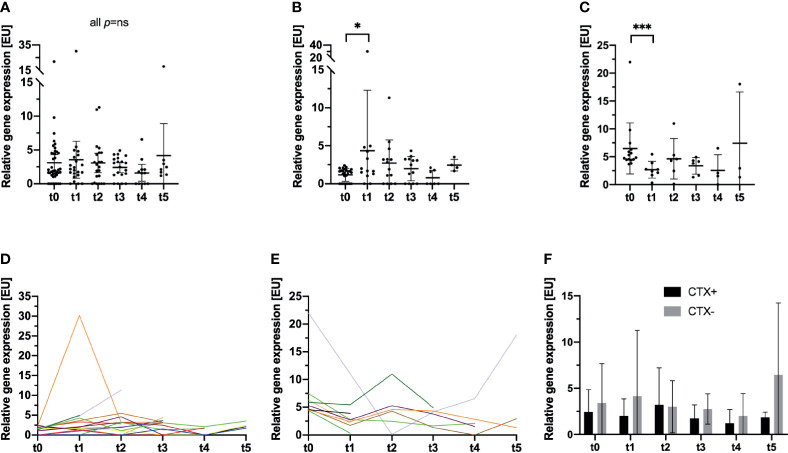
Longitudinal analysis of blood samples of 47 CRC patients for the incidence and relative enumeration of CTC by a semi-quantitative CK20 RT-qPCR. Results are expressed by expression units [EU]. The bar represents the mean relative CTC count expressed by [EU]. **(A)** The entire study cohort is analyzed, and no statistically significant deviations of the relative CTC count compared to t0 were monitored. **(B–E)** The previously reported clinically significant cut-off value for CTC detection by CK20 PCR in CRC patients (13) was applied and the cohort stratified for further analysis. **(B, D)** In patients, who were below the cut-off at t0, a significant increase in CTC at t1 was monitored (p=0.047). No further differences were monitored at later visits compared to t0. Each color represents an individual patient. **(C, E)** For patients who were above the cut-off at t0, a significant decrease in the relative CTC quantity was recorded (p<0.001). No further significant deviations were monitored at later visits. **(F)** Patients were stratified and subdivided according to their necessity of adjuvant chemotherapeutic treatment (CTX+). Patients that did not require adjuvant treatment were grouped in the follow-up subset (CTX-). *p≤0.05; ***p≤0.001.

In line with the findings obtained with the NYONE^®^ cell imager, no statistically significant short-term effect on the CTC/CK20-positivity load by the surgical procedure could be observed (mean: 3.11 [EU], SD: 3.81 at t0, and mean: 3.55 [EU], SD: 6.17 at t1; p not significant). Like the cytological analysis with the NYONE^®^ cell-imager, the RT-qPCR analysis did also not reveal any significant alteration of the relative CTC/CK20-positivity throughout the study period compared to the pre-operative CTC signal at t0 (all p not significant) (**Table 2** and **Figure 3A**).

Next, we further stratified patients by applying a clinically and prognostically relevant cut-off for relative CTC positivity by CK20 RT-qPCR, which was established in a previous study (13). Patients were divided into two subgroups pre-operatively exhibiting either a high CTC positivity (≥2.77 [EU]; CTC-high) or a low CTC positivity (<2.77 [EU]; CTC-low). Patients who were pre-operatively (t0) in the CTC-low group, post-operatively (t1) showed a statistically significant increase in CTC numbers measured by CK20 RT-qPCR (mean: 1.17 [EU], SD: 0.85 at t0 *vs*. 4.36 [EU], SD: 7.94 at t1; p=0.047) (**Figures 3B, D**) which then declined again until t4 (mean: 0.78 [EU], SD: 0.99). Interestingly, a statistically significant increase in the relative CTC/CK20-positivity could be monitored from 9 to 12 months after surgery (t4 to t5; mean: 0.78 EU, SD: 0.99 at t4 *vs*. mean: 2.44 EU, SD: 0.77 at t5; p=0.018).

In contrast, in patients with a pre-operative high CTC-positivity, the surgical procedure and hence tumor burden reduction significantly reduced the CTC load during the first month (mean: 6.49 EU, SD: 4.56 at t0 *vs*. 2.68 EU, SD: 1.51 at t1; p<0.001). However, analyses at later time points throughout the study then revealed a slight increase of CTC compared to the post-operative CTC load at t1 (**Figures 3C, E**).

Interestingly, comparing the data obtained at t1 of both patient subsets, no difference emerged in the relative quantity of CTC between the groups (mean: 2.68 [EU], SD: 1.51 at t1 CTC high and mean: 4.36 [EU], SD: 7.94 at t1 CTC low; p=ns). Patients with pre-operative high CTC counts dropped post-operatively to a comparable level of patients with pre-operative low CTC counts who exhibited a post-operative increase in relative CTC loads.

Next, we subdivided the patients examined by CK20 RT-qPCR according to their status of adjuvant chemotherapy analogs to the cohort of patients in the NYONE^®^ subset. Contrary to the obtained data of the NYONE^®^ subgroup analysis, CTX seemed to have an effect on the CTC enumeration. Patients in the CTX+ cohort showed lower relative CTC counts by trend at almost all re-visits. Only at t2 was there a slightly higher CTC count in patients of the CTX- subgroup (CTX+: mean 3.21 [EU], SD 3.98 *vs*. CTX-: mean 3.00 [EU], SD 2.81; p=ns) (**Table 2** and **Figure 3F**)

### Evaluation of Individual Longitudinal Patient Courses

After having analyzed the overall cohort, we next focused on patients´ individual CTC courses detected by the two enumeration methods and linking the obtained results to the clinical follow-up data.

**Figure 4:**
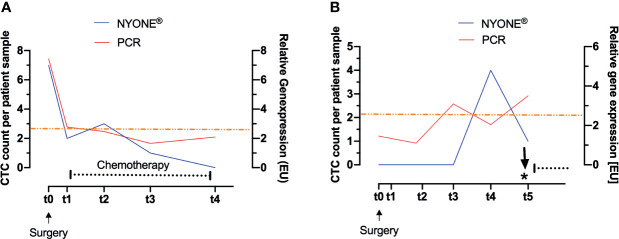
Evaluation of CTC load during individual longitudinal patient courses. CTC were enumerated by two altering methods: cytologically after IF-staining by detection with the NYONE^®^ (blue line) and molecularly by analysis of CK20-gene expression by RT-qPCR (red line). The asterisk indicates the time point of diagnosis of the local recurrence of carcinoma. The black dotted line indicates the interval of chemotherapy. The orange line represents the clinically significant cut-off value of CTC detected by CK20 RT-qPCR. **(A)** CRC patient with stage III carcinoma of the descending colon. After the operation, the CTC load dropped markedly and under the adjuvant therapy, no significant rise in CTC was observed. **(B)** CRC patient with stage II carcinoma of the descending colon. A total of 24 weeks after the operation, a clear rise of CTC in both detection methods was monitored, with the PCR-based approach being even earlier Clinically no significant follow-up event was recorded. Thirteen months after t0 a local recurrence of the carcinoma was diagnosed.

**Figure 4A** exemplarily displays the context and interplay of CTC enumeration and adjuvant treatment after surgery. This patient was diagnosed with an adenocarcinoma of the descending colon, but with locally progressed tumor burden and nodal positive stage III disease. In line with the guidelines (German S3-Guideline Colorectal Carcinoma, Version 2.1 – January 2019 AWMF-Registration Number: 021/007OL) the patient was admitted to adjuvant chemotherapy after surgical resection. Fitting to the advanced tumor burden, the patient showed exceptionally high numbers of CTC pre-operatively (t0) (NYONE^®^: 7 cells, PCR: 7.44 [EU]) in comparison to the general average of the cohort. Around 1 month (t1) after surgery and shortly before initiation of chemotherapy, the CTC count had dropped significantly (NYONE^®^: 2 cells, PCR: 2.77 [EU]) and remained below the cut-off values of both detection methods during the entire course of adjuvant chemotherapy (t1 up to t3) and the end of the observation period (t5). However, even though CTC enumeration from t3 until t5 was below the cut-off value, the PCR-based approach revealed a slight increase in the CTC enumeration starting at t4, at which the cytological approach further indicated a decline in the CTC load. Tumor markers CEA and CA19.9 were monitored at t0 and were not elevated at that time. Follow-up diagnostics were performed within the investigation period and did not show any signs of macroscopical tumor relapse. Since the patient dropped out of the study after t5, we could not further elaborate whether the CTC increase determined indirectly by CK20 RT-qPCR was indicative of a reactivation of a minimal residual disease (MRD) and clinical relapse.

**Figure 4B** exemplarily displays the disease course of a patient diagnosed with a stage II adenocarcinoma of the descending colon and provides an example of the potential of CTC as biomarkers for add-on recurrence diagnostics. According to the general guidelines (German S3-Guideline Colorectal Carcinoma, Version 2.1 – January 2019 AWMF-Registration Number: 021/007OL), adjuvant chemotherapy was not given, and the patient was only admitted to oncological follow-up. Pre-operatively (t0), no CTC could be detected with the NYONE^®^ cell imager and the relative enumeration by CK20 RT-qPCR also revealed a measurement below the cut-off value. At t2 (3 months later), CTC detection by both the NYONE^®^ and PCR was still negative, and the PCR-based analysis even revealed a slight decrease of the CTC count (1.46 [EU] at t0 and 1.10 [EU] at t2). At t3 (6 months later), a relevant increase of the relative CTC enumeration above the cut-off value could be monitored by CK20 RT-qPCR (3.09 [EU]), while the NYONE^®^ analysis still did not reveal any CTC positivity. At t4 (9 months after surgery), a significant increase in the CTC count could also be detected by the microscopical enumeration approach (0 cells at t0-t3 and 4 cells at t4). The last measurement at t5 (12 months after surgery) revealed a continuous increase in the relative quantification of CTC by CK20 RT-qPCR (3.50 [EU]), but surprisingly a declining cytological detection by the NYONE^®^ (4 cells at t4 and 1 cell at t5). Interestingly, 13 months after initial surgery, a local recurrence of the primary adenocarcinoma was detected. Important to note that the tumor markers CEA and CA19.9 were below the cut-off levels at all times. In summary, these findings suggest that we were able to monitor a significant increase in CTC by both techniques being indicative of a reactivation of an MRD prior to its detection by the imaging diagnostics conducted according to the standard guidelines of follow-up (German S3-Guideline Colorectal Carcinoma, Version 2.1 – January 2019 AWMF-Registration Number: 021/007OL).

## Discussion

The benefit of CTC diagnostics as a biomarker for assessing the disease prognosis in cancer patients is evident (6, 9). However, the majority of studies firstly focus on patients with systemic stage IV disease and, secondly, conclude the patients’ prognosis by CTC-analysis from a single pre- or post-operative blood sample only. Accordingly, CTC numbers or CTC associated parameters were determined only once and correlated with the clinical follow-up (16–21). Only very few studies have analyzed the prognostic potential of longitudinal CTC quantification over a period of time (22, 23).

In this prospective pilot study, we longitudinally monitored a cohort of CRC patients of miscellaneous tumor stages, who underwent surgical resection of the tumor and postoperatively were either admitted to oncological follow-up or adjuvant chemotherapy treatment. All patients enrolled were recontacted for serial blood samples, partially on the occasion of routine follow-up examination over a course of 12 months post-operatively. CTC enumeration was carried out by two differing techniques, (i) cytologically, by IF staining and microscopical detection by the semi-automated cell imager (NYONE^®^), and (ii) molecularly, by a semi-quantitative RT-qPCR detecting CK20 mRNA as an epithelial cell marker. CTC enumeration data obtained by either method was correlated with clinical characteristics and follow-up data.

Firstly, contrary to our expectations, surgery did not have any statistically significant effect on the quantity of CTC detected by the cytological approach utilizing the cell imager NYONE^®^ regarding the overall complied cohort of our study population. Generally, and described by Galizia et al. (24), one could expect the resection of the tumor to have a significant negative impact on the incidence of CTC postoperatively in the overall cohort. Despite this, we were able to demonstrate a significant short-term increase in CTC quantity post-operatively but only in patients who were pre-operatively negative for CTC in the cytological method or below the cut-off in the PCR-based analysis. During the surgical procedure and mechanical manipulation of the tumor, an excess of CTC may be released into the bloodstream (25–27). Owing to a short half-life of CTC, their numbers are significantly reduced but still elevated 3 months after surgery compared to the preoperative status. Interestingly, in patients who pre-operatively showed evidence for CTC in the NYONE^®^ or were above the cut-off for the CK20 RT-qPCR method, CTC levels post-operatively dropped significantly. Concluding, the removal and physical manipulation of the tumor in this subset of patients did surprisingly lead to a significant decrease in CTC numbers in the short term. In a large single-center study comprising 403 patients with breast cancer, van Dalum et al. analyzed CTC with the CellSearch^®^ system over a long follow-up period (median 5.7 years). In their study, they did not observe any relevant impact of the surgical procedure on CTC frequency. Enumeration levels were fairly constant over the study period (28). Then again, CTC are often thought to be directly linked to the primary tumor. Hence, once the tumor is resected, the general opinion would be that CTC numbers are decreasing and due to CTC clearance ultimately disappear from peripheral blood samples. Our data suggest that CTC remain detectable even in the mid and long-term after the surgical procedure. Notable in this context is the exceedingly high CTC count of 29 IF positive cells in one patient twelve months post-operatively. Interestingly, this was a patient who was diagnosed with a colon cancer of the descending colon with a singular hepatic metastasis who underwent synchronous resection of the primary tumor and the metastasis. Then, the patient received an adjuvant chemotherapy and CTC counts remained low throughout the study period. The blood draw at t5 was during the routine visit of oncological follow-up. Unfortunately, the patient then dropped out for further analysis.

Presumably, these CTC are shed into the bloodstream and are derived from disseminated tumor cells (DTC) that rest in a dormant-like stage in the bone marrow or lymph nodes. When suitable triggers are active, they initiate local recurrence or macro-metastasis.

In a xenograft mouse model and co-culture experimental set-up, Möhrmann et al. demonstrated the importance of DTC and their potential to act as a source for tumor relapse (29). In a study of breast cancer patients, Meng et al. showed that in 13 of 36 patients, where follow-up data were available, CTC were detectable up to 22 years after treatment. As they concluded slowly replicating or dormant DTC to be the cause of this (30), it is reasonable to assume that the origin of CTC detectable in our patients after surgical removal of the tumor are DTC or derivatives of these.

Furthermore, our data further underscore the potential of CTC as prognostic biomarkers in CRC, which has been already shown in previous studies (11–13). By applying our CK20 RT-qPCR on blood samples over the post-operative course of the disease, we were able to detect a relative rise in CTC ahead of clinical symptoms or positive radiological imaging in a patient with stage II colon carcinoma who was diagnosed with local tumor recurrence thirteen months after tumor resection (**Figure 4A**). In other malignancies, for instance, in leukemia patients, the concept of molecular minimal residual disease (MRD) monitoring is well established (31). PCR methods for detection of genes or genetic aberrations for MRD monitoring have been standardized by the Europe Against Cancer (EAC) consortium and are widely instituted (32). In terms of colorectal cancer as a common solid tumor entity, concepts comparable to the MRD monitoring in leukemia are still lacking. The follow-up is mainly conducted by clinical examinations and imaging diagnostics according to the general guidelines (German S3-Guideline Colorectal Carcinoma, Version 2.1 – January 2019 AWMF-Registration Number: 021/007OL). In this study, we demonstrate an example where disease monitoring by CK20 RT-qPCR based CTC detection is feasible and plausible. Even though no clinical recommendation can be drawn based on our data yet, attention should be given to the unambiguous case report of the stage II colon cancer patient reported on above.

In our study, we were able to identify patients with unusual CTC courses. The initial blood draw, revisit and correlation of the data with clinical follow-up characteristics of some patients allowed for individual insights into the prognostic potential and relevance of CTC. Again, the vast majority of studies focus on the overall potential of CTC as a biomarker in cancer patients. Larger patient cohorts are recruited, and the relevance of CTC enumeration is correlated to clinical follow-up data. Though, for further analysis of the principles of changes over time in the CTC quantity, further in-depth analysis possibly also investigating the biology of these individual CTC are desirable.

Future investigations on the prognostic potential of CTC in the follow-up analysis of CRC patients should aim at a multi-marker approach. As a widely employed and well-esteemed technique for CTC detection, CellSearch^®^ (Menarini Silicon Biosystems) is up to date the only method approved by the FDA (Food and Drug Administration) and therefore commercially available for clinical application. Here, CTC are enriched and enumerated by an immunological antibody-based method, which has been described in detail elsewhere (33). The CellSearch^®^ technique utilizes antibodies targeting two antigens: EpCAM and EGFR. The clinical significance had been documented firstly by Cohen et al. in a large prospective study, though patients enrolled were all diagnosed with stage IV disease (15). It is the general opinion that a high tumor burden with distant metastasis (stage IV disease) correlates with high numbers of CTC, hence the detection of CTC by those two markers as applied by CellSearch^®^ is presumably more likely leading to significant numbers of CTC. Only very few studies were conducted analyzing non-metastatic CRC patients and the incidence of CTC by CellSearch^®^ detection. A possible explanation could be the concise enumeration rates of CTC detected by CellSearch^®^. As presented by Thorsteinson et al. in their study analyzing the prognostic relevance of CTC detection by CellSearch^®^ in non-metastatic patients, the detection rate of CTC is poor (34), though the samples size in terms of the number of patients recruited was quite small. Another investigation by Gazzaniga et al., in which high-risk non-metastatic CRC patients were enrolled, led to similar results of low CTC numbers detected and a lack of correlation with clinical characteristics or efficacy as a prognostic marker (35). Perchance, the sensitivity of the CellSearch^®^ system is limited due to only two markers being applied, and hence the rate of undetected CTC is high.

Therefore, to overcome this potential pitfall, we intended to establish a strategy employing a multi-marker approach extending the utilized range of markers EpCAM and EGFR by two further broadly established epithelial markers: pan-CK and HER2. However, despite broadening the range of applied markers for detection, the rate of CTC was also low in our study. A possible explanation for this could be the process of Epithelial-Mesenchymal-Transition (EMT). CTC that have undergone this process may have lost or downregulated such epithelial antigens (36, 37). Consequently, these CTC were missed by IF staining for our markers and thus not detected. Few studies have been published analyzing appropriate mesenchymal marker antigens for CTC detection in CRC patients. Yokobori et al. for example, have identified the actin-bundling protein Plastin 3 by microarray analysis of a cohort of CRC patients and demonstrated its negative prognostic value in a large patient cohort (38). In future efforts, the significance and potential of mesenchymal antigens have to be further validated. An approach for evading the issue of epithelial- and mesenchymal-specific detection of CTC could be the label-free isolation and enumeration. In general, CTC are thought to be significantly larger (>8 µm) than leucocytes, allowing for the concept of isolation by size of epithelial tumor cells (ISET) (39, 40). One way of conducting CTC enumeration by ISET is the filtration of blood samples through a porous membrane, allowing leucocytes to pass and CTC to be effectively retained on the membrane as it has been exemplarily demonstrated with the ScreenCell^®^ isolation devices (ScreenCell^®^, Sarcelles, France). Staining of these CTC then allows for cytological analysis and enumeration. The feasibility and prognostic value have been demonstrated (18, 41, 42).

In summary, our study enlightens the kinetics of CTC in CRC patients after resection of the primary tumor and provides data concerning the CTC quantity over a long-term follow-up. This study not only supports the significance of CTC as a prognostic biomarker but also provides a more in-depth longitudinal analysis of CTC over the course of the disease. Furthermore, these data suggest that by using CK20 RT-qPCR for CTC detection and enumeration approach (e.g., during long-term follow-up), a molecular MRD monitoring might be feasible in CRC patients allowing earlier detection and therapy decision making in relapse situations. However, future investigations with an even more extended follow-up and larger patient cohorts will have to validate our results and may help to define an optimal longitudinal sampling scheme for liquid biopsies in the post-operative monitoring of cancer patients to enable tailored therapy concepts for precision medicine.

## Data Availability Statement

The original contributions presented in the study are included in the article/supplementary material. Further inquiries can be directed to the corresponding author.

## Ethics Statement

The studies involving human participants were reviewed and approved by ethics committee of the UKSH Campus Kiel and the Medical Faculty, University of Kiel. The patients/participants provided their written informed consent to participate in this study.

## Author Contributions

Conceptualization: AH, BB, RG, CR., SH, and SS. Data curation: AH, BB, and RG. Formal analysis: AH, KD, BB, and SS. Investigation: AH. Methodology: KD, BB, and RG. Project administration: SH and SS. Resources: BB, CR, CS, TB, SH, and SS. Software: BB and RG. Supervision: SH and SS. Validation: BB. Visualization: AH. Writing—original draft: AH and SS. Writing—review and editing: AH, KD, BB, RG, CR, CS, TB, SH, and SS. All authors contributed to the article and approved the submitted version.

## Conflict of Interest

At the time of the study, RG was working for ORGA Labormanagement GmbH, which provided a CTC test using NYONE^®^. She is now working for SYNENTEC GmbH, which produces and distributes the NYONE^®^ imaging system. 

The remaining authors declare that the research was conducted in the absence of any commercial or financial relationships that could be construed as a potential conflict of interest.
